# The Ministry of Health and Sanitation, Sierra Leone  – Public Health England (MOHS-PHE) Ebola Biobank

**DOI:** 10.12688/wellcomeopenres.15279.2

**Published:** 2019-10-30

**Authors:** Bernie Hannigan, Jimmy Whitworth, Miles Carroll, Allen Roberts, Christine Bruce, Thomas Samba, Foday Sahr, Elizabeth Coates

**Affiliations:** 1 Public Health England, London, SE1 8UG, UK; 2 London School of Hygiene & Tropical Medicine, London, WC1E 7HT, UK; 3 Ministry of Health and Sanitation, Freetown, Sierra Leone; 4 University of Sierra Leone, 34th Military Hospital, Freetown, Sierra Leone; 5 University of Sierra Leone, Freetown, Sierra Leone

**Keywords:** Ebola, biobank

## Abstract

During the Ebola outbreak in 2014-2015 in Sierra Leone, residual clinical specimens and accompanying data were collected from routine diagnostic testing in Public Health England (PHE) led laboratories. Most of the samples with all the accompanying data were transferred to PHE laboratories in the UK for curation by PHE.  The remainder have been kept securely in Sierra Leone. The biobank holds approximately 9955 samples of which 1108 tested positive for Ebola virus. Researchers from the UK and overseas, from academia, government other research organisations and commercial companies can submit proposals to the biobank to access and use the samples.

The Ministry of Health and Sanitation in Sierra Leone (MOHS) retains ownership of the data and materials and is working with PHE and other researchers to develop and conduct a series of research projects that will inform future healthcare and public health strategies relating to Ebola.  The Ebola Biobank Governance Group (EBGG) was established to guarantee equality of access to the biobank for the most scientifically valuable research including by researchers from low and middle-income countries. Ensuring benefit to the people of Sierra Leone is an over-arching principle for decisions of the EBGG.  Four ongoing research collaborations are based on the first wave of biobank proposals approved by EBGG.  Whilst the biobank is a valuable resource its completeness and sample quality are consistent with the outbreak conditions under which they were collected.

## Disclaimer

The views expressed in this article are those of the author(s). Publication in Wellcome Open Research does not imply endorsement by Wellcome.

## Introduction

During the Ebola virus disease outbreak in 2013–2015 in Sierra Leone ( https://www.who.int/features/ebola/storymap/en/), Public Health England (PHE) operated three diagnostic laboratories: in Freetown (Kerrytown), North West Sierra Leone (Port Loko Laboratory) and Central Sierra Leone (Makeni laboratory). These laboratories processed up to 300 clinical samples each day during the outbreak. In 2015 approximately 9955 residual samples and associated data were collected from these laboratories and transferred to PHE in the UK leading to the establishment of the MOHS-PHE Ebola Biobank with the approval of the Sierra Leone Government. The biobank has been funded by a Wellcome Trust Bioresource (grant reference: 208376).

PHE acts as the curator of the samples on behalf of the Sierra Leone government. The MOHS retains ownership of the data and samples and is working with PHE and other researchers to enable research to inform future healthcare and public health strategies relating to Ebola.

To our knowledge, this was the first biobank to be established to enable the sharing of samples from this outbreak with the global research community however a 2016 review by the WHO indicates significant other holdings.’ See https://www.who.int/blueprint/priority-diseases/key-action/biobanking_ebola_samples/en/

## The MOHS-PHE Ebola Biobank Governance and Access Processes

This biobank is a secure but accessible resource of biological samples that are essential for understanding human disease and the development of vaccines, diagnostics and treatments. The biobank samples are an especially valuable and finite resource, a legacy of the world’s largest ever outbreak of Ebola virus disease https://www.who.int/features/ebola/storymap/en/. Outbreaks of this disease continue in Africa, so it is evident that further biomedical research is required to more readily bring outbreaks under control https://www.who.int/emergencies/diseases/ebola/drc-2019.

The existing materials are also a record of other diseases that were incident in the population concurrently with Ebola.

The MOHS-PHE Ebola Biobank Governance Group (EBGG) was established to address ethics and governance associated with the establishment and running of the biobank, focussing on realising the clinical and scientific value of the materials. The Group includes three members from Sierra Leone nominated by the Chief Medical Officer. Other members of the group represent the World Health Organisation, the Wellcome Trust, the UK Department for International Development and PHE with the Chair from the London School of Hygiene and Tropical Medicine. 

The EBGG guarantees equality of access to the materials and data for researchers from developed as well as low- and middle-income countries. It ensures that the resource is used for the global public good in accordance with the undertaking given by PHE to the MOHS, and that the research will have relevance to the people of Sierra Leone. 

The biobank is accessible to all researchers globally from academia, government other research organisations and commercial companies. Because the samples were collected during an outbreak—where the primary objectives were to deliver care and limit the outbreak—ethical approval for research was not collected from patients. So, in addition to EBGG approval, researchers wishing to use its samples need to obtain ethical approval from the Sierra Leone national ethics committee and from a local institutional ethics committee prior to receiving their samples. Researchers can only access fully anonymised samples which are released with a material transfer agreement that guarantees the rights of the Sierra Leone government to any intellectual property developed during the research study and ensures the publication of all research results. 

## Samples and associated data

The 9955 samples comprise whole blood, blood plasma and swabs; 1108 of these tested positive for Ebola virus. Laboratory results are available for malaria testing, but not for other diseases that may have afflicted Ebola virus disease patients. All biobank samples are stored in freezers at −80°C.

A database was established at PHE to store the associated data. Not all the samples have a full dataset, but the information in Box 1 is available for some samples in the biobank

**Box 1. Details of information available for samples**

**Table:** 

• Laboratory of origin	• Date of hospitalisation
• Laboratory ID number	• Symptom onset
• Facility from where the patient was referred	• Date tested
• Patient age	• Clinical chemistry results
• Gender	• Ebola viral load
• Original or follow up sample	• Malaria test result ^1^
• Ebola test result ^2^	

^1^ Diagnosis of Malaria was by rapid diagnostic test kits.

^2^ Diagnosis of Ebola was by real-time PCR.

Currently there are no patient outcome details, but it is expected that this information will be available for inclusion in the database in the future.

Usually most samples are collected for inclusion in biobanks in accordance with protocols designed to guarantee consistency and high quality. They are also handled, processed and stored in ideal conditions. As the MOHS-PHE biobank comprises residual materials that were used for diagnosis there is variation in sample quality. Prior to their movement to PHE facilities, the samples may have been subjected to different storage conditions depending upon available facilities. Some had to be transported long distances to centres for testing and may have been subjected to cycles of freezing and thawing. The conditions the samples have been held in since their arrival in the UK in 2015 are fully documented. Samples are provided to researchers with the caveat that their quality cannot be guaranteed.

Variability in the quality of the diagnostic assays used during the outbreak has led to some samples being incorrectly classified and many of the samples may contain other undiagnosed pathogens. All samples are assumed to be highly infectious and will only be released, without further treatment, to researchers with documented access to Biosafety Level 4 facilities when all the appropriate permits are in place. Samples may be released when the risk of them containing live virus has been eliminated using a validated inactivation procedure. The method of virus inactivation is discussed with the receiving researcher and currently has involved the use of a commercial RNA/DNA extraction kit. We plan to use X-ray radiation for inactivation at some point in the future.

## Applying to the MOHS-PHE Ebola Biobank

The EBGG seeks to collaborate with bona fide researchers wishing to undertake research that is in the public good and that will inform future healthcare and public health strategies in Sierra Leone. Application to the biobank is a one stage process made to the MOHS-PHE EBGG.

The biobank is a limited resource so research projects for which the scientific value has been scrutinized through peer review organised by a known organisation are prioritised. The amount and nature of depletable sample required is considered carefully and must be justified within the application in relation to the intended use. The EBGG secretariat maintains contact with the recipient research groups to ensure the materials are used for the intended health-related purposes and the Group will assess if the research use meets the required criteria for access (including legal and ethics standards). Researchers will also need to show that they have sufficient funding to cover the cost of preparing and delivering samples as well as research costs. For applications involving researchers from low and middle-income countries, this criterion may be waived. Applicants wishing to access materials are advised to contact the MOHS-PHE EBGG secretariat’ to confirm sample availability before their project plans are finalised.

## Current research

The following research collaborations are ongoing:

*Colonel Professor F. Sahr, Military Hospital, Freetown, Sierra Leone & Dr Felicity Fitzgerald UCL*

**The identification of the pathogens underlying febrile illnesses in children attending Ebola Holding Units testing negative for Ebola to identify opportunities for improving clinical outcomes**

Stored plasma from children who tested negative for Ebola will be tested to identify pathogens in children with febrile illnesses. Understanding of the epidemiology of the commonest pathogens will allow the ruling out of Ebola infection and the delivery of targeted therapy. It will also inform future public health planning.

*Professor Michael Levin & Dr Nathalie MacDermott, Imperial College London*

**Exploring the genetic factors that play an important role in susceptibility, severity and outcome from infectious disease**

There is growing evidence that exposure to Ebola results in different outcomes, from contacts remaining uninfected to severe, fatal infection. Among survivors some recover fully, others have virus persistence and others develop long term complications such as arthritis and uveitis. Identification of the genetic basis for susceptibility severity and outcome of Ebola will provide further information on its pathogenesis and new targets for therapy.

*Dr Christopher H. Logue & Ms Emma Wise, PHE*

**Comparison of the viromes (collection of viruses) of Ebola negative and Ebola positive patients to establish the role that non-VHF pathogen co-infection may play in affecting clinical outcome and survival.**

Significant developments have been made in the understanding the biology of Ebola but there remain gaps in our knowledge of the effects of co-infection with pathogens that co-circulate during an Ebola outbreak, and the role that pathogen co-infection may play in affecting clinical outcome and survival remains largely unknown. We hypothesize that the clinical outcome of patients infected with Ebola was detrimentally affected by co-infection with other circulating pathogens. 

*Dr Robert Watson, PHE*

*Ebola MoDRAD*

**The development and delivery of a rapid and bedside diagnostic tool to significantly increase the capacity to handle future Ebola outbreaks**

The overall aim of Ebola MoDRAD is to develop and deliver rapid and bedside diagnostic tool(s), by way of a multidisciplinary research consortium drawn from key European and African research organisations.

The requested samples will be used to improve and validate the following rapid point-of-care diagnostics for use in the field:

- Validation of an Ebola blood inactivation tube for serology.
- IgM (IgG) antigen microarray epitope mapping of patient sera
- Validation of an Ebola antigen / IgM antibody lateral flow device
- Validation of rapid point-of-care isothermal assay

## The future of the MOHS-PHE Ebola Biobank

PHE’s work includes a commitment to support research into the effective management of future disease outbreaks. The MOHS-PHE Ebola Biobank is unique in the UK in being housed in facilities suitable for the storage and processing of high-risk biological samples that require specialist handling by highly trained competent staff in appropriate Advisory Committee on Dangerous Pathogens hazard group 4 laboratories. Within these facilities, protocols are used for the inactivation of Ebola virus and other high-risk pathogens potentially contained in the samples, so they may be shared with researchers globally who lack access to such facilities. Therefore, the governance, management, laboratory processing and sharing of biobank resources have been detailed and documented. They can be re-employed by PHE or any other relevant organisation dealing with specimens that might be collected during any future infectious disease outbreak.

So, while the current biobank holdings are intended to be depleted by sharing with researchers, its laboratory and contextual legacy will be sustained. PHE is continuing to explore additional innovative and efficient ways to ensure researcher safety when dealing with highly dangerous viral pathogens so that high quality research may proceed. Additionally, planning is underway to include biobank operation and management as an element of laboratory training for Sierra Leone staff who may then more effectively undertake investigations on residual diagnostic samples. 

## Data availability

No data are associated with this article.

