# Supplementary material for: The Ministry of Health and Sanitation, Sierra Leone  – Public Health England (MOHS-PHE) Ebola Biobank
Source: Wellcome Open Res. 2019 Oct 30;4:115. Originally published 2019 Aug 1. [Version 2] doi: 10.12688/wellcomeopenres.15279.2 (PMC6737994; doi:10.12688/wellcomeopenres.15279.2)
Supplement: Peer review history

# Peer review: Reviewer response for version 1

**DOI:** 10.21956/wellcomeopenres.16677.r36191

**Published:** 2019-09-10

**Authors:** Edgar Simulundu

**Affiliations:** 1 Department of Disease Control, University of Zambia, Lusaka, Zambia

**General comments**

 The manuscript by Hannigan *et al*. describes the creation and use of an Ebola biobank based on samples collected in Sierra Leone during the 2013-2015 West African Ebola virus disease outbreak. The manuscript describes a very important bio-resource with potential for significantly advancing viral haemorrhagic fever research and improving public health. The manuscript is generally well written, but there are some areas that can be improved. Specific comments that may require the attention of authors are given below:

**Specific comments**

**Title**

 I am of the view that the title should be modified to read something like “Establishment of Ebola Biobank based on the West African Outbreak”.

**Abstract**

 It is better for the abstract to have a single paragraph.

 To avoid repetitions, in the sentence “Most of the samples with all the accompanying data were transferred to PHE…” should be modified to read “Most of the samples with all the accompanying data were transferred to PHE laboratories in the UK for curation”.

 I suggest the sentence “Researchers from the UK and overseas,…” be rephrased to read “Researchers globally…”.

 The sentence “Four ongoing research collaborations are based on the wave of biobank proposals approved by EBGG” appears to occur without some background. It is better to indicate to the readership something like “So far, the EBGG has approved four research proposals…”.

 It would be better to place a comma after the word “resource” in the sentence “Whilst the biobank is a valuable resource its completeness…”.

**Introduction**

 The Introduction should have some reference(s) relating to the 2013-2015 Ebola outbreak to allow some readers who may be interested or lack the background information about this outbreak to gain deeper insight of this epidemic.

 It would also be better to mention (along with an appropriate reference) of the availability of other Ebola biobanks, which are publicly accessible. If such a biobank is not available, an indication that this is the first of its kind would strengthen this section.

 It is better to have a comma after the year 2015 in this sentence “In 2015 approximately…”.

**Establishment of the MOHS-PHE Ebola Biobank**

 In this section, the above title is a bit misleading in that what is described is some background on the West African Ebola outbreak and the establishment of the Ebola Biobank Governance Group (EBGG). Furthermore, the governance and modalities of accessing the samples are described. Meanwhile, information regarding the actual establishment of the biobank has not been described. I would suggest that this section be divided into two sections: one that describes how the biobank was established and the other which describes the governance and modalities of accessing and utilization of the biobank.

 It is better to place a comma after the word “secure” in the sentence “This biobank is a secure but accessible…”

 An appropriate reference is needed for this sentence “a legacy of the world’s largest ever outbreak of Ebola virus disease”. Some readers would be interested in the scale of other Ebola virus outbreaks.

 It would be better to support the following sentence with a reference: “Outbreaks of this disease continue in Africa,…”. An article describing the current ongoing Ebola outbreak in the Democratic Republic of Congo could be used.

**Samples and associated data**

 The following sentence can be revised to improve clarity “Results are available for malaria testing but there are no results available for testing for other diseases that may have been carried by patients”. Authors can consider something like “Laboratory results are available for malaria testing, but not for other diseases that may have afflicted Ebola virus disease patients”.

 In the sentence “All biobank samples are held in freezers at −80°C,” the word “held” can be replaced with the word “stored.”

 I suggest that the whole of the sentence “Until then it is possible for researchers to infer patient outcome by looking at sequential diagnostic tests” be deleted. Such inferences may be erroneous.

**Applying to the MOHS-PHE Ebola Biobank**

 Being a co-owned biobank, it would be better that applicants contact an overarching body rather than just PHE as suggested by this sentence “Applicants wishing to access materials are advised to contact PHE to confirm sample availability before their project plans are finalised”.

I confirm that I have read this submission and believe that I have an appropriate level of expertise to confirm that it is of an acceptable scientific standard.

# Peer review: Reviewer response for version 1

**DOI:** 10.21956/wellcomeopenres.16677.r36095

**Published:** 2019-08-22

**Authors:** Mark Stephen Lever

**Affiliations:** 1 CBR Division, Defence Science and Technology Laboratory (Dstl), Salisbury, UK

This letter describes the establishment of the MOHS-PHE Ebola Biobank, the potential benefits, current studies and proposed future for the samples.

 The rationale and benefits of such a facility are made clear, particularly in the current climate where additional scrutiny and accountability are important factors to be considered. This letter clearly sets out the establishment of the Ebola Biobank Governance Group to oversee the equality of access to the samples for all researchers. This letter achieves the aim of advertising such access, for the scientific community, and citing the ongoing research which is benefitting from the samples.

 For additional completeness, it might be useful to add to this letter any previous research collaborations and their objectives that have now finished.

I confirm that I have read this submission and believe that I have an appropriate level of expertise to confirm that it is of an acceptable scientific standard.
